# A prospective evaluation of cardiovascular magnetic resonance measures of dyssynchrony in the prediction of response to cardiac resynchronization therapy

**DOI:** 10.1186/s12968-014-0058-0

**Published:** 2014-08-01

**Authors:** Manav Sohal, Simon G Duckett, Xiahai Zhuang, Wenzhe Shi, Matthew Ginks, Anoop Shetty, Eva Sammut, Sebastian Kozerke, Steven Niederer, Nic Smith, Sebastien Ourselin, Christopher Aldo Rinaldi, Daniel Rueckert, Gerald Carr-White, Reza Razavi

**Affiliations:** 1 Division of Imaging Sciences and Biomedical Engineering, Kings College London, London, UK; 2 The Department of Cardiology, Guy’s and St Thomas’ NHS Foundation Trust, London, UK; 3 Centre for Medical Image Computing, University College London, London, UK; 4 Biomedical Image Analysis Group, Department of Computing, Imperial College London, London, UK; 5 Division of Imaging Sciences, The Rayne Institute, 4th Floor, Lambeth Wing, St Thomas’ Hospital, London SE1 7EH, UK

**Keywords:** Cardiac resynchronization therapy, Cardiovascular magnetic resonance, Dyssynchrony, Heart failure, Strain, Regional volume-change SDI

## Abstract

**Background:**

Many patients with electrical dyssynchrony who undergo cardiac resynchronization therapy (CRT) do not obtain substantial benefit. Assessing mechanical dyssynchrony may improve patient selection. Results from studies using echocardiographic imaging to measure dyssynchrony have ultimately proved disappointing. We sought to evaluate cardiac motion in patients with heart failure and electrical dyssynchrony using cardiovascular magnetic resonance (CMR). We developed a framework for comparing measures of myocardial mechanics and evaluated how well they predicted response to CRT.

**Methods:**

CMR was performed at 1.5 Tesla prior to CRT. Steady-state free precession (SSFP) cine images and complementary modulation of magnetization (CSPAMM) tagged cine images were acquired. Images were processed using a novel framework to extract regional ventricular volume-change, thickening and deformation fields (strain). A systolic dyssynchrony index (SDI) for all parameters within a 16-segment model of the ventricle was computed with high SDI denoting more dyssynchrony. Once identified, the optimal measure was applied to a second patient population to determine its utility as a predictor of CRT response compared to current accepted predictors (QRS duration, LBBB morphology and scar burden).

**Results:**

Forty-four patients were recruited in the first phase (91% male, 63.3 ± 14.1 years; 80% NYHA class III) with mean QRSd 154 ± 24 ms. Twenty-one out of 44 (48%) patients showed reverse remodelling (RR) with a decrease in end systolic volume (ESV) ≥ 15% at 6 months. Volume-change SDI was the strongest predictor of RR (PR 5.67; 95% CI 1.95-16.5; P = 0.003). SDI derived from myocardial strain was least predictive. Volume-change SDI was applied as a predictor of RR to a second population of 50 patients (70% male, mean age 68.6 ± 12.2 years, 76% NYHA class III) with mean QRSd 146 ± 21 ms. When compared to QRSd, LBBB morphology and scar burden, volume-change SDI was the only statistically significant predictor of RR in this group.

**Conclusion:**

A systolic dyssynchrony index derived from volume-change is a highly reproducible measurement that can be derived from routinely acquired SSFP cine images and predicts RR following CRT whilst an SDI of regional strain does not.

## Background

Cardiac resynchronization therapy (CRT) is established as an effective treatment in selected heart failure patients with evidence of dyssynchrony, improving both morbidity and mortality [1]. Despite advances in imaging and device technology, the clinical non-responder rate has remained static at approximately 30% [2], whilst the proportion failing to demonstrate left ventricular (LV) reverse remodeling (RR) is closer to 40-50% [3]. QRS duration (QRSd) is the most widely utilized marker of dyssynchrony but it is only a moderate predictor of CRT response [4] and does not always correlate with mechanical dyssynchrony [5]. The mechanics of myocardial contraction and relaxation are complex with multiple methods currently used to investigate dyssynchrony. This has led to extensive, but ultimately unsuccessful, work in the field of echocardiography to develop imaging based mechanical dyssynchrony measures that improve patient selection [3]. It is not entirely clear if the concept of mechanical dyssynchrony is flawed or if the most widely used method of measuring it (echocardiography) is the limiting factor. There are inherent limitations with echocardiography such as sub-optimal image quality and associated problems with reproducibility and these may impact the integrity of any data acquired. Additionally, analysis methods based on echo Doppler largely look at two-dimensional longitudinal motion and this may over-simplify the complex mechanisms involved in myocardial contraction. Newer echocardiographic techniques that use speckle tracking to measure strain have been advocated as better methods of measuring mechanical dysynchrony and CRT response but this has yet to be demonstrated in a multi-center setting [6],[7].

Cardiovascular magnetic resonance (CMR) provides superior image quality and complete LV coverage, [8] as well as information about the position and extent of myocardial scar [9] and detailed cardiac anatomy (including that of the coronary veins) [10]. Measures of dyssynchrony, similar to those derived from echocardiography, can be derived from cine and tagged images, [11],[12] looking at volume change, myocardial thickening and strain (radial, circumferential or longitudinal) [12],[13]. Recent studies have shown that CMR imaging combining scar quantification and measures of strain can predict response to CRT [14]. In this study, we set out to use CMR to comprehensively investigate the mechanics of LV contraction. Measures of mechanical dyssynchrony represented by volume change, myocardial thickening and strain were evaluated as predictors of CRT response alongside the degree of left ventricular scarring and relation to LV lead position.

## Methods

The study had two phases:

Phase 1 – Comprehensive evaluation of CMR based measures of mechanical dyssynchrony to determine the optimal predictor(s) of CRT response.

Phase 2 – Application of the optimal predictor(s) identified in phase 1 to a second cohort of patients and comparison with accepted predictors of CRT response (QRS duration, LBBB morphology and scar burden) [15],[16].

### Phase 1

#### Study population and initial assessment

Forty-four patients fulfilling standard criteria for CRT (drug refractory heart failure, LVEF ≤35% and prolonged QRS > 120 ms) were prospectively recruited from a dedicated pre-assessment clinic for patients being considered for CRT in our institution. The local ethics committee (Westminster) approved the study and informed consent was obtained from each patient. Patients completed a quality-of-life questionnaire (QOL) and six-minute walk as well as standard 2D echocardiogram to assess LV volumes pre- and six-months post CRT. Echocardiographic measures of dyssynchrony were also measured. To assess dyssynchrony the inter-ventricular mechanical delay (IVMD) was calculated as the difference between the LV and right ventricular (RV) pre-ejection periods measured from QRS onset to the onset of pulmonary and aortic flows respectively [17]. To measure previously reported parameters for intra-ventricular dyssynchrony, we used TDI. We calculated the difference between septal to lateral peak velocity within the aortic valve opening (AVO) and closing times (AVC). Real-time transthoracic 3D echocardiography [18] (RT3DE) was performed on all patients and volumes analysed with TomTec 4D LV-Analysis software (TomTec Imaging systems Inc, Munich, Germany).

Echocardiograms were acquired on a GE vivid 7 scanner (General Electric-Vingmed, Milwaukee, Wisconsin). Analysis was performed using EchoPac version 6.0.1 (General Electric-Vingmed). Ejection fractions (EF) and LV volumes were measured using the 2D biplane Simpson’s method.

#### CMR acquisition

Patients were scanned using 1.5 T MR-scanner (Achieva, Philips Healthcare, Best, Netherlands) with a 32-element cardiac coil. The CMR framework described below was also performed on 10 healthy volunteers to assess the reproducibility of the measured variables. Cardiac synchronization was performed with vector electrocardiography. After localization and a coil sensitivity reference scan, an interactive real-time scan was performed to determine cardiac geometry in the short axis (SA), 4-chamber (4CH), 3-chamber (3CH) and 2-chamber (2CH) orientations. A multiple slice cine steady state free precession (SSFP) scan was performed in a stack of SA slices covering the LV and in the 4CH, 3CH and 2CH orientations (FA = 60°, TR/TE = 2.9/1.5 ms, resolution 2.2×2.2×10 mm, 30 heart phases). In addition, a SA and long-axis stack of breath hold complementary modulation of magnetization (CSPAMM) cine images or 3D CSPAMM [19] images (three breath holds each 18 heart beats, tagged spacing 7.7 mm, 14 heart phases, TR/TE 6.9/3.2 ms) were acquired of the whole LV. Late gadolinium enhancement (LGE) multi-slice IR gradient echo sequence imaging (FA = 25°, TR/TE = 5.8/2.0 ms) was performed 15-20 mins following the administration of 0.1-0.2 mmol/kg gadopentetate dimeglumine (Magnevist®, Bayer Healthcare, Dublin, Ireland) using conventional inversion recovery techniques to delineate areas of scar [20]. Where scar was identified, the amount was quantified and expressed as a percentage of myocardium using CMR^42^ software (Circle Cardiovascular Imaging Inc, Calgary, Canada).

#### Methods for CMR processing

**Figure 1:**
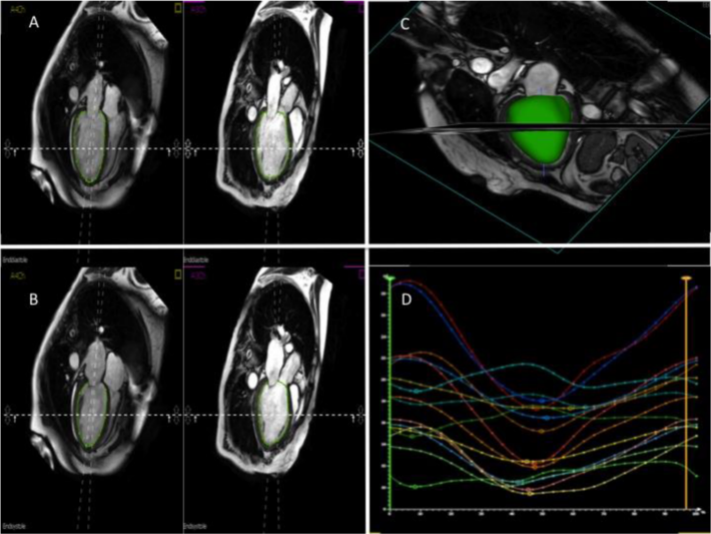
**Representative figure from the TomTec platform used to determine volume-change SDI.(A and B)** The software requires endocardial contours to be outlined at end-diastole and end-systole in the 4-chamber, 3-chamber and 2-chamber (not shown). The software then produces a 3-dimensional shell of the endocardial cavity that tracks the endocardial contours throughout the cardiac cycle **(C)**. Time-volume curves are then generated that represent the time to reach minimum volume for each of the 16 segments of the LV **(D)**.

The regional volume change within the LV cavity over the cardiac cycle for 16 segments (defined using the American Heart Association model of the LV) was determined using TomTec 4D LV-Analysis software (TomTec Imaging systems; Unterschleissheim, Germany). The software performs semi-automatic segmentation and propagation of the LV endocardial border from the SA stack and three long axis SSFP cine images (Figure 1).

**Figure 2:**
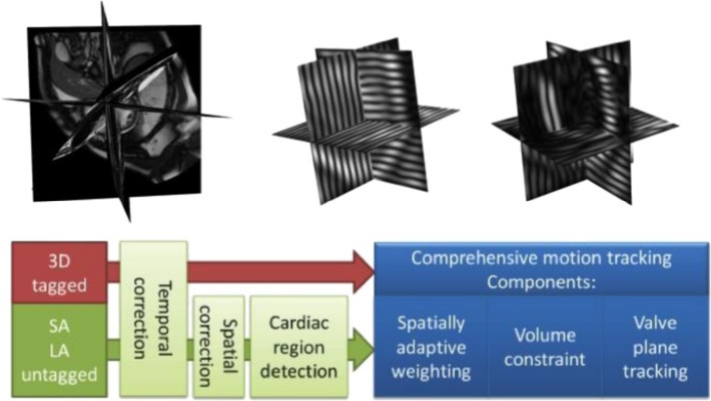
**Workflow of the CMR processing framework.** Data from untagged and tagged MR images are combined and then subject to temporal and spatial correction before comprehensive motion tracking is computed.

**Figure 3:**
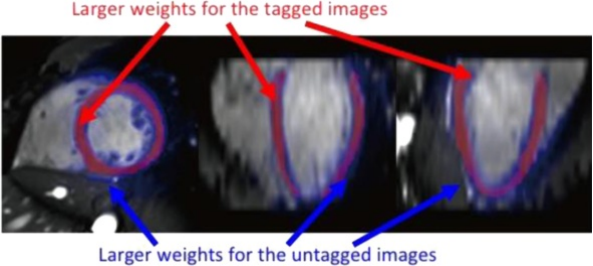
**Differential weighting of the endocardial and epicardial regions and myocardial regions from tagged and untagged images.** More weight is given to the untagged cine images when assessing endocardial and epicardial motion (blue arrows) whilst more weight is given to the tagged images when assessing myocardial motion (red arrows).

To quantify myocardial strain and wall thickening, we developed a framework (Figure 2), which semi-automatically computes these values at each phase across the cardiac cycle [21],[22]. The framework included the following steps:

(1) Manual slice-by-slice segmentation of the endocardial and epicardial borders of the LV in the stack of SA and three long-axis SSFP images in the first end diastolic (ED) cardiac phase.

(2) Spatial re-positioning of the SA and long-axis SSFP and CSPAMM slices, to correct for any misalignment due to the different breath-hold positions by mutual rigid registration of the segmented SSFP endocardial and epicardial borders and CSPAMM slices.

(3) Identification of the mitral valve plane from the insertion point of the valve leaflets in the first ED cardiac phase of the three long-axis images.

(4) Deformation of a 3D atlas based model of the LV to the manually delineated endocardial and epicardial segmentations and mitral valve plane [21].

(5) Deformable registration of the subsequent SSFP and CSPAMM phase of the cine images to the first ED phase and the extraction of deformation fields within the myocardium of the 3D heart model for myocardial motion and strain computation. Stronger weighting was given to the registration information from SSFP images for calculation of the deformation of the endocardial and epicaridal borders and from the registration information from CSPAMM images for the deformation within the body of the myocardium (Figure 3). We used serial propagation registration to calculate the large deformation field required for the registration between the phases that were far from the ED phase. In the serial propagation, we first registered the neighbouring phase of the ED phase to the reference image. The resulting transformation was used to initialize the registration of the next phase. This process continued until all phases were registered with the reference image.

(6) The propagated endocardial and epicardial borders were checked against the SSFP images to ensure accuracy of the borders and the mitral valve plane throughout the cardiac cycle.

(7) The myocardial wall thickening values were computed from the surface distance between the endocardial and epicardial surfaces for each of the 16 segments of the left ventricular myocardium.

(8) Myocardial strain was computed from the deformation field of the myocardium as follows. Having the deformation fields *v(x,p)* for voxel *x*, from the ED phase to phase *p*, the myocardial motion was:

(1) $$m\left(x\right)=\left|v\left(x,p\right)\right|$$

Strain was then calculated from the motion in the radial, circumferential and longitudinal directions for each voxel and all the values within each of the 16 segments of the myocardium were averaged to calculate overall strain as well as strain in each of the three directions.

#### Systolic dyssynchrony index (SDI)

The SDI was calculated for regional cavity volume change, maximum muscle thickness and peak strain. The SDI was defined as the standard deviation (SD) of the regional times to peak volume change, maximum muscle thickness or peak strain. The SDI was expressed as a percentage of the cardiac cycle to allow for heart rate variation.

#### Implant procedure and characterization of LV lead position in relation to scar

Patients underwent CRT implant with the LV lead positioned in a lateral or posterolateral position wherever possible. Where venous anatomy restricted lead positioning to a non-lateral/posterolateral position, the choice of LV lead position was at the preference of the operator. Right ventricular and right atrial leads were implanted as per operator preference. The lead position relative to scar was determined using a modified clock-face method [23]. Briefly, in the LAO projection, the mitral valve annulus was pictured as a clock face with 12–2 o’clock representing an anterior position, 2–4 o’clock a lateral position, 4–5 o’clock a posterolateral position and 5–6 o’clock a posterior position. In the RAO projection, the long axis of the LV was divided into basal, mid and apical segments. CRT implant fluoroscopy was reviewed to score the LV lead position and compare it to areas of LGE on CMR.

#### Classification of reverse remodelling and clinical response

Patients were deemed to have reverse remodeled (RR) if there was a ≥ 15% reduction in LV end-systolic volume (ESV) as measured using the modified Simpson’s biplane method on 2D echo images [2]. Evaluation of symptomatic response was made by assessing NYHA class, six-minute walk distance and QOL score. Patients were labeled clinical responders if they met 2 out of 3 of the following criteria: ≥10% improvement in six-minute walk distance; NYHA class reduction of ≥ 1; ≥20% reduction in QOL score.

#### Comparison and reproducibility of methods

Volume SDI (derived from TomTec 4D LV-Analysis software) intra- and inter-observer agreements were assessed according to the statistical methods proposed by Bland and Altman [24]. The process to develop muscle thickening and strain SDI is an automatic process so intra-observer or inter-observer assessment is not appropriate. The accuracy of manually drawn end-diastolic contour propagation was assessed using a 1 to 5 scoring system, with 5 being perfect tracking and 1 inaccurate tracking.

#### Statistical analysis

Statistical analysis was performed on PASW Statistics 20 (SPSS Inc, Chicago, IL, USA). A Shapiro-Wilk test was used to ensure variables were normally distributed. Continuous variables were expressed as mean ± SD. Nominal variables were expressed as absolute count and percentages and compared with a Fisher’s exact test. The optimal cut-off for each dyssynchrony measure to predict CRT response was determined from receiver-operator characteristic (ROC) curves. Adjusted prevalence ratios (PR) of response were calculated for each measure using Poisson regression with robust standard errors to determine the strength of each measure as a predictor of CRT response [25]. The same method was used to identify the quantity of scar that was associated with an adverse response to CRT.

### Phase 2

Having identified the optimal CMR-derived measure of dyssynchrony, a second cohort of patients was studied to assess the applicability of that measure to a different patient population. Each patient met standard criteria for CRT as per phase 1 and underwent identical clinical and echocardiographic assessment pre-CRT and at 6-month follow up. CMR image acquisition was abbreviated from that performed in phase 1 on the basis that the optimal measure of dyssynchrony could be measured from standard SSFP cine images. Accordingly, no CSPAMM or 3D CSPAMM images were acquired. For the same reason, the full CMR imaging processing algorithm to extract strain and thickening measures was not performed in the second cohort of patients.

#### Comparison with other predictors of response

QRS duration ≥150 ms, LBBB morphology and the degree of scar/fibrosis have been identified as predictors of CRT response [16],[26]. The utility of the optimal CMR-derived predictor of response to predict CRT response was tested in a multivariate model including the aforementioned predictors.

## Results

Forty-four patients were recruited to the first phase of the study (91% male, mean age 63.3 ± 14.1 years, 80% NYHA class III), and 50 patients for the second phase (70% male, mean age 68.6 ± 12.2 years, 76% NYHA class III). The clinical characteristics of patients studied in phase 1 and phase 2 are shown in Table 1. Patients in phase 2 tended towards a shorter mean QRS duration but this was not statistically significant (146 ± 21 ms vs 154 ± 24; P = 0.11). The phase 2 cohort had significantly more females (30% vs 9%: P = 0.01). Both groups were matched in terms of etiology, number of patients with scar (and mean scar burden), baseline NYHA class, medical therapy and baseline LV volumes derived from 2D echocardiography. Similarly, there was no significant difference in LV lead position between the two groups. The proportion of patients with their LV lead in regions with >50% transmural scar was not significantly different between the two groups (9% cohort 1 vs 14% cohort 2; P = 0.46).

**Table 1:** Characteristics of patients in phase 1 and phase 2

	**Phase 1 cohort**	**Phase 2 cohort**	**P value**
**N**	44	50	
**Age (years)**	63.3 ± 14.1	68.6 ± 12.2	0.06
**Sex (male/female)**	40/4	35/15	0.01
**Etiology**	23 DCM	31 DCM	0.34
	21 ICM	19 ICM	
**Number of patients with scar on CMR n(%)**	24 (55)	21 (42)	0.22
**Scar burden on CMR in those patients with scar (% of myocardium)**	21.4 ± 7.9	24.4 ± 11.4	0.35
**QRS duration (ms)**	154 ± 24	146 ± 21	0.11
**QRS morphology (LBBB/RBBB/IVCD)**	31/5/8	28/4/18	0.15
**Rhythm**	38 SR	42 SR	0.53
	6 AF	8 AF	
**Beta blockers n(%)**	40 (91)	38 (76)	0.11
**ACEI/ARB n(%)**	43 (98)	49 (98)	0.84
**Diuretics n(%)**	27 (62)	35 (70)	0.44
**Aldosterone antagonists n(%)**	17 (38)	33 (66)	0.27
**NYHA class n(%)**			
**II**	7 (16)	12 (24)	0.23
**III**	35 (80)	38 (76)	
**IV**	2 (4)	0	
**QOL score pre CRT**	51 ± 24	50 ± 26	0.41
**6 minute walk distance (m)**	255 ± 112	295 ± 149	0.02
Ejection fraction (%)*	25 ± 9	22 ± 9	0.14
End diastolic volume (ml)*	232 ± 72	218 ± 90	0.43
End systolic volume (ml)*	175 ± 67	172 ± 82	0.88
**LV lead position**			0.86
**Lateral**	20	21	
**Posterolateral**	21	22	
**Anterior**	3	5	
**Posterior**	3	2	
**LV lead in area with >50% transmural scar n(%)**	4(9)	7(14)	0.46

*Volumes and ejection fraction derived from 2D echocardiography.

*Abbreviations*: *CMR* cardiovascular magnetic resonance, *LBBB* left bundle branch block, *RBBB* right bundle branch block, *IVCD* non-specific interventricular conduction delay, *AF* atrial fibrillation, *ACEI* angiotensin converting enzyme inhibitor, *ARB* angiotensin receptor blocker, *NYHA* New York Heart Association, *QOL* quality of life.

### Phase 1

#### Reverse remodeling and clinical response

Pre-implant LVESV and LVEF were 175 ± 67 ml and 25 ± 9% respectively. These improved over the follow-up period to 155 ± 68 ml (P = 0.03) and 31.8 ± 10.2% (P < 0.001). RR was seen in 21 out of 44 patients (48%). Thirty-six (82%) patients had a reduction of at least one NYHA class at 6 months, 38 (86%) had a ≥ 20% reduction in quality of life score. Twenty-eight patients (64%) increased their 6-minute walk distance by 10% or more. Using the pre-determined definition of a clinical responder, 36 (82%) were deemed clinical responders to CRT.

#### CMR measures of dyssynchrony predicting reverse remodelling (Table 2, Figure 4)

**Table 2:** Receiver-operating characteristic analysis of the CMR-derived predictors assessed using the novel framework

	**AUC**	**Cut-off**	**Sensitivity**	**Specificity**
**Volume change SDI**	0.85	9.75%	0.85	0.82
**Thickening SDI (>0.5 mm)**	0.75	15.8%	0.65	0.79
**Radial strain SDI**	0.59	22.2%	0.95	0.29
**Longitudinal strain SDI**	0.59	20.7%	0.83	0.40
**Circumferential strain SDI**	0.6	8.9%	0.60	0.62
**Combined strain SDI**	0.58	5.6%	0.80	0.46

*Abbreviations*: *AUC* area under the curve, *SDI* systolic dyssynchrony index.

The area under the curve (AUC) statistic, optimal cut-off values, sensitivities and specificities for each method to predict reverse remodeling (RR) is shown.

**Figure 4:**
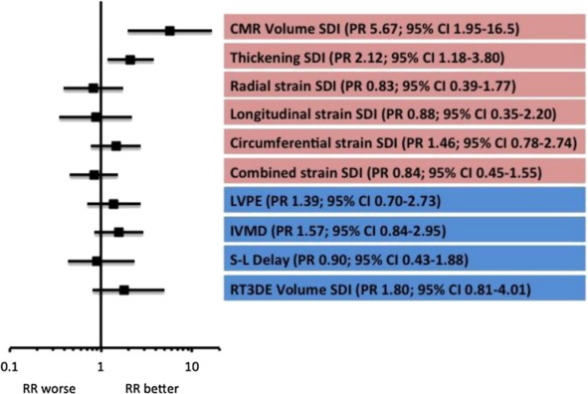
**Univariate forest plot comparing the CMR-derived dyssynchrony measures and echocardiography-derived measures as predictors of RR in phase 1.** The red boxes indicate CMR-derived measures and the blue indicate echo-derived measures. Abbreviations: LVPE – left ventricular pre-ejection; IVMD – interventricular mechanical delay; S-L delay – septal to lateral wall delay; RT3DE volume SDI – volume SDI derived from real-time 3D echo.

**Figure 5:**
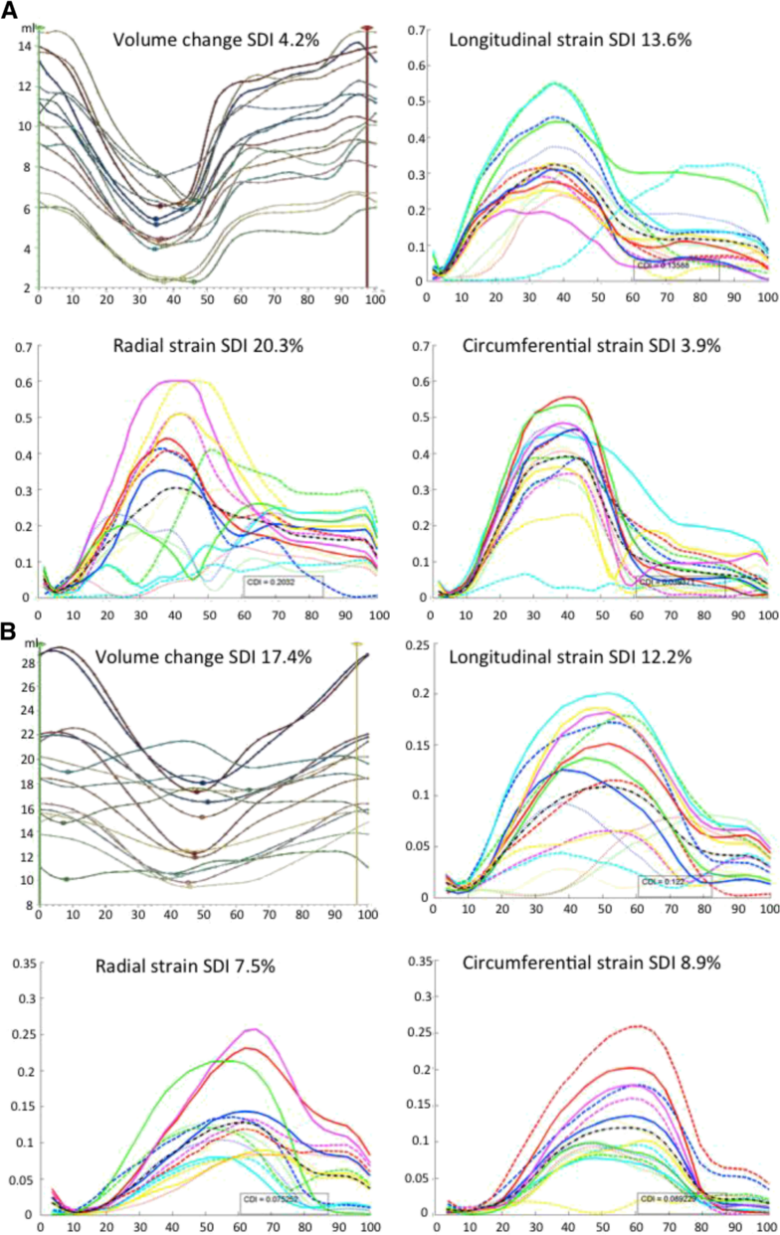
**Volume-change and strain SDI curves from a healthy volunteer (A) and heart failure patient with LBBB and QRS duration of 200 ms (B).** The volume change curves for the healthy volunteer are congruous indicating synchrony whilst the strain curves are not perfectly congruous. The volume change curves for the heart failure patient show clear dyssynchrony whereas the strain curves suggest synchrony.

Table 2 shows the results of the ROC analysis for each of the CMR-derived measures. The optimal cut-off for volume-change SDI was >9.75% (sensitivity 85% and specificity 82%) and was highly predictive of RR (PR 5.67; 95% CI 1.95-16.5; P = 0.003). The SDI of myocardial thickening was predictive of RR but the association was not as strong as that for volume-change SDI (PR 2.12; 95% CI 1.18-3.80). None of the strain measures were predictive of RR. Figure 4 charts how well the CMR-derived indices predicted reverse remodeling when dichotomized according to the optimal cut-offs identified. Figure 5 gives an example of the volume-change and strain curves from a healthy volunteer and also from a patient with heart failure, LBBB and QRS duration of 200 ms. In the healthy volunteer the curves for volume-change SDI were very congruous (with associated low SDI), indicating synchronous contraction, whilst those for strain were less so (with relatively high SDIs). The volume SDI curves for the patient with heart failure demonstrate marked dyssynchrony (volume change SDI 17.4%) but the longitudinal, radial and circumferential strain SDI curves are not inidicative of marked dyssynchrony.

#### The effect of scar

LGE imaging for scar was possible in 43 patients. Twenty-four patients had scar and the mean scar burden in this group was 21.4 ± 7.9% of myocardium. The cut-off for predicting lack of reverse remodeling was 14.7% with a sensitivity of 91% and specificity of only 50%. This level of scar or greater was a predictor of attenuated RR response (PR 0.51; 95% CI 0.27-0.98; P = 0.044). Four patients were identified as having their LV lead position in a position of scar. Of these, none demonstrated RR at 6 months.

#### Comparison with echocardiographic measures of dyssynchrony

The four echocardiographic measures of dyssynchrony were evaluated as predictors of RR. A forest plot comparing the echocardiographic measures and CMR-derived measures is shown in Figure 4. Volume SDI derived from RT3DE was the best echocardiographic measure but it did not predict RR as well as MRI-derived volume change SDI (PR 1.80, 95% CI 0.81-4.01 for RT3DE volume change SDI versus PR 5.00, 95% CI 1.72-14.56 for MRI-derived volume change SDI).

#### Reproducibility

Twenty patients and 10 volunteers were selected for intra and inter-observer variability for volume SDI. In healthy controls the intra-observer average difference was 0.06 ± 0.4% and coefficient of variation (COV) was 2.0 ± 1.1% and the inter-observer average difference was 0.40 ± 1.2% and COV was 3.1 ± 2.6%. In CRT patients, the intra-observer average difference was 0.20 ± 0.5% and the COV was 2.8 ± 0.9%. The inter-observer average difference was 0.70 ± 1.0% and the COV 3.6 ± 3.0%.

The mean accuracy score for the endocardial and epicardial segmentation propagation was 3.89 ± 0.89% (a score of 5 indicating perfect tracking and a score of 1 inaccurate tracking).

### Phase 2

#### Reverse remodeling and clinical response

**Figure 6:**
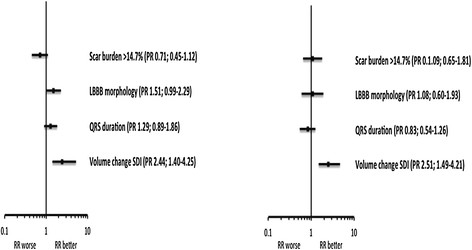
**Forest plots of the utility of volume-change SDI to predict RR in phase 2 compared to the presence of scar, LBBB morphology and QRS duration.** The panel on the left is the univariate model and the panel on the right is the multivariate model.

RR was seen in 35 (70%) of patients and clinical response (as defined by meeting 2 out of 3 of the NYHA, QOL and 6-minute walk distance criteria) was seen in 42 (84)%. The differences in clinical characteristics between RR responders and non-responders are shown in Table 3. Responders were more likely to have underlying LBBB (66% versus 33% for non-responders; P = 0.04). There was a trend towards patients with scar being more likely to non-respond although this did not reach statistical significance. There was no difference in LV lead position between the groups although pacing the LV in regions of scar was associated significantly more common in non-responders (3% responders vs 47% non-responders: P < 0.001). Having identified volume-change SDI as the best predictor of RR in the first cohort, a cut-off of 9.75% was applied to the second patient population to further explore the utility of volume-change SDI as a predictor of RR. Volume-change SDI was predictive of RR (PR = 4.44; 95% CI 1.78-12.4; P = 0.001). In total, 29 patients had a volume-change SDI >9.75% and of these 27 (93%) reverse remodeled. Of the 21 patients with an SDI <9.75%, only 8 (38%) reverse remodeled (P < 0.001). In a multivariate regression model including QRSd ≥150 ms, QRS morphology, scar burden >14.7% and volume-change SDI, volume change SDI was the only statistically significant independent predictor of reverse remodelling (Figure 6).

**Table 3:** Characteristics of responders vs non responders – Phase 2

	**Responders**	**Non-responders**	**P value**
**N**	35	15	
**Sex (male/female)**	23/12	12/3	0.31
**Aetiology**	24 DCM	7 DCM	0.14
	11 ICM	8 ICM	
**QRS duration (ms)**	149 ± 20	141 ± 23	0.27
**QRS morphology (LBBB/RBBB/IVCD)**	23/1/11	5/3/7	0.04
**Number of patients with scar on CMR n(%)**	12 (34)	9 (60)	0.11
**Scar burden on CMR (% of myocardium) in those patients with scar**	22.2 ± 13.0	29.4 ± 8.4	0.17
**Volume change SDI (%)**	15.2 ± 6.2	8.5 ± 4.5	<0.001
**LV lead position**			0.20
**Lateral**	17	4	
**Posterolateral**	14	8	
**Anterior**	2	3	
**Posterior**	2	0	
**LV lead in area with >50% transmural scar n (%)**	1(3)	7(47)	<0.001

Abbreviations: As for Table 1.

## Discussion

The purpose of our study was to use comprehensive CMR assessment to deliver better insights into the complexities of cardiac mechanical dyssynchrony in heart failure whilst exploring the relationship to CRT response. Different measures of mechanical dyssynchrony were assessed using the same 16 regional segment framework of the LV so as to understand and compare the effectiveness of each measure in predicting chronic RR to CRT.

The main findings were:

1) An SDI of ≥ 9.75% derived from volume change was sensitive, specific and superior to other mechanical dyssynchrony measures for predicting chronic reverse remodeling. In multivariate analysis it was the only significant independent predictor of RR post-CRT.

2) An SDI derived from peak strain (radial, longitudinal, circumferential or combined) was poor at predicting RR.

Increasing the response rate to CRT has been the focus of many studies and the results to date have been conflicting. QRS duration remains the most widely utilised marker of dyssynchrony and it has the advantage of being relatively easy to measure and highly reproducible. Historic guidelines advocated the use of QRSd >120 ms as a cut-off for CRT but the importance of more prolonged QRSd (≥150 ms) and QRS morphology (LBBB) in predicting response has been reflected in the most recent iterations of international guidelines for CRT implant [26]. In our study, even when including QRSd ≥ 150 ms and LBBB morphology as covariates in a multivariate model, volume-change SDI remained the only statistically significant predictor of RR post CRT in the phase 2 cohort.

Many studies have focused on the use of echocardiographic measures to predict response. The failure to deliver robust, reproducible measures is well documented [3] and this is partly due to problems with ‘noise’ that has affected inter and intra observer variability. An advantage of the methods described in this study is that the CMR data was acquired in standardised fashion and post-processing was carried out using automated software which therefore precluded test, re-test variation. This is reflected in the favourable reproducibility analyses reported for the current study.

A striking finding was the failure of strain measures to predict response. Although surprising, considering the focus of previous studies on measures of myocardial strain [13],[14], this apparent separation of volume measures of dyssynchrony from strain indices may be explained by the mechanics involved in myocardial contraction. Ejection is due to wall thickening and apical-basal shortening. In a normal subject, wall thickening is the result of a combination of shearing and elongation along the laminar sheets that make up the myocardium [27] and has a limited correlation with fibre shortening. Fibre shortening is more closely related to measures of strain [28]. The use of longitudinal, radial and circumferential strain dyssynchrony measurements that are not aligned with the orientation of the helical cardiac microstructure [29] has physiological limitations. In the healthy heart there is intrinsic variation in the time taken for different regions to contract and reach their peak strain and this may obscure any changes in synchrony that may occur with heart failure. This variation is significant with a time to peak circumferential strain variation of 121 ms [30] in the human left ventricle. The distribution of timing to peak deformation is consistent with the distribution of electrical activation [31] only amplified [32]. This increased heterogeneity in the spatial variation in deformation is potentially facilitated by multiple factors including the non-uniformity of fibre and sheet orientation [33]. Therefore, using indices of strain to measure myocardial motion may not provide a useful measure of dyssynchrony and may explain why these measures were not useful in predicting which patients were likely to respond to CRT. This point is reinforced when assessing healthy volunteers with normal ventricles (Figure 5). The volume SDI in that normal volunteer was 4% indicating synchrony, but the strain curves were quite incongruous even though the LV was normal. Using more global measures of mechanical dyssynchrony such as volume SDI, gives a much clearer indication of how well coordinated the ventricle is contracting as it does not take into account the different forces working with and against each other during systole. Part of the aim of the study was to differentiate muscle thickening and strain derived SDI, which are more related to active myocardial contraction, with volume change, which combines active and passive myocardial motion (as seen in regions of scar). In patients with significant scar, it is known that position and extent of myocardial scar affect the likelihood of response to CRT [34] and in our phase 2 cohort only 34% of patients with significant scar demonstrated RR. However, in the multivariate model, volume derived SDI was an independent predictor of RR even when taking the presence of significant scar into account.

### Limitations

The main limitations of the study are the relatively small sample size and the follow-up duration of only 6 months. The fact that CMR cannot be used for follow up studies with most current CRT devices is worth considering as the use of change in ESV measured using echo as an end-point also has some limitations. It is important to acknowledge the history of echocardiographic measures of dyssynchrony which appeared promising in single-centre studies but which were then found to be less robust and reproducible when investigated in a multi-centre study [3] The data from the current study demonstrate the need for an appropriately powered multi-centre randomised-controlled trial to evaluate the broad applicability of CMR-derived volume change SDI as a predictor of CRT response.

## Conclusion

Volume-change SDI is easily derived from the most commonly run cine sequences and strongly predicted response in patients receiving CRT whilst measures of dyssynchrony based on regional strain did not. At a time when increasing numbers of heart failure patients are undergoing evaluation with CMR (given its excellent resolution and tissue characterisation) the addition of this novel predictor of CRT response may be of potential value.

### Clinical perspective

The literature is replete with studies designed to identify better (mainly echocardiographic) predictors of CRT response. The role of mechanical dyssynchrony as a discriminator for patient selection has been strongly challenged and most contemporary CRT guidelines focus on the importance of the electrical substrate, with the strongest recommendations reserved for those with the widest QRS and LBBB. The superior imaging afforded by CMR has not been comprehensively evaluated till now and in 2 independent cohorts of patients meeting conventional CRT criteria we have shown that CMR-derived volume change SDI is a superior predictor of RR than both QRS duration and LBBB morphology. We acknowledge the importance of learning lessons from studies evaluating dyssynchrony measures derived from echocardiography and the findings of this study need to be further assessed in a larger, multi-centre setting. If our findings are confirmed then we would contend that patients meeting criteria for CRT implant should receive conventional CRT if their volume change SDI is >9.75% and that the group with SDI <9.75% should be evaluated more closely. Alternative means of delivering CRT, including multi-site and LV endocardial pacing, may provide superior options in this group (many of whom have an ischaemic aetiology and have areas of scar) [35].

## Abbreviations

CRT: Cardiac resynchronization therapy

LV: Left ventricle

RR: Reverse remodelling

CMR: Cardiovascular magnetic resonance

LBBB: Left bundle branch block

EF: Ejection fraction

QOL: Quality of life

SA: Short axis

SSFP: Steady state free precession

CSPAMM: Complementary modulation of magnetization

ED: End diastolic

SDI: Systolic dyssynchrony index

ESV: End systolic volume

EDV: End diastolic volume

NYHA: New York Heart Association

ROC: Receiver operating characteristic

PR: Prevalence response

## Competing interests

Dr Manav Sohal, receives research funding from St. Jude Medical.

Dr Matthew Ginks, receives research funding from St. Jude Medical.

Dr Anoop Shetty, receives research funding from St. Jude Medical.

Dr C Aldo Rinaldi, is a Consultant to St. Jude Medical and receives investigator led grant funding from St Jude Medical and Medtronic Inc.

Professor Reza Razavi, receives investigator led grant funding from Philips Healthcare.

## Authors’ contributions

MS, SGD, CAR, GCW and RR conceived and designed the study. MS and SDG performed the data analyses and MS drafted the manuscript. XZ, WS, SK, SN, NS, SO and DR designed the image processing framework. MG, AS and ES were responsible for clinical data collection. All authors read and approved the final manuscript.
